# Development of a Biomimetic Chondroitin Sulfate-modified Hydrogel to Enhance the Metastasis of Tumor Cells

**DOI:** 10.1038/srep29858

**Published:** 2016-07-19

**Authors:** Yang Liu, Shujun Wang, Dongsheng Sun, Yongdong Liu, Yang Liu, Yang Wang, Chang Liu, Hao Wu, Yan Lv, Ying Ren, Xin Guo, Guangwei Sun, Xiaojun Ma

**Affiliations:** 1 Scientific Research Center for Translational Medicine, Dalian Institute of Chemical Physics, Chinese Academy of Sciences, Dalian, 116023, China; 2 Department of Biotechnology, Dalian Institute of Chemical Physics, Chinese Academy of Sciences, Dalian, 116023, China; 3 University of Chinese Academy of Sciences, Beijing 100049, China; 4 College of Life Science & Bioengineering, Beijing University of Technology, Beijing, 100124, China; 5 School of Life Science, Dalian University, Dalian, 116023, China; 6 Dalian Municipal Central Hospital, Dalian, 116033, China

## Abstract

Tumor metastasis with resistance to anticancer therapies is the main cause of death in cancer patients. It is necessary to develop reliable tumor metastasis models that can closely recapitulate the pathophysiological features of the native tumor tissue. In this study, chondroitin sulfate (CS)-modified alginate hydrogel beads (ALG-CS) are developed to mimic the *in vivo* tumor microenvironment with an abnormally increased expression of CS for the promotion of tumor cell metastasis. The modification mechanism of CS on alginate hydrogel is due to the cross-linking between CS and alginate molecules via coordination of calcium ions, which enables ALG-CS to possess significantly different physical characteristics than the traditional alginate beads (ALG). And quantum chemistry calculations show that in addition to the traditional egg-box structure, novel asymmetric egg-box-like structures based on the interaction between these two kinds of polymers are also formed within ALG-CS. Moreover, tumor cell metastasis is significantly enhanced in ALG-CS compared with that in ALG, as confirmed by the increased expression of MMP genes and proteins and greater *in vitro* invasion ability. Therefore, ALG-CS could be a convenient and effective 3D biomimetic scaffold that would be used to construct standardized tumor metastasis models for tumor research and anticancer drug screening.

Tumor metastasis with resistance to anticancer therapies is the main cause of death in cancer patients^1^. The molecular mechanisms underlying tumor metastasis are still not well known, and chemotherapy has a limited impact on improving the survival rate. Therefore, it is necessary to develop reliable tumor metastasis models that can closely recapitulate the pathophysiological features of the native tumor tissue and its surrounding microenvironment, which would be beneficial for understanding the mechanisms of tumor metastasis and testing chemotherapeutics^2^.

A three-dimensional (3D) model based on a 3D scaffold is the subject of recently increasing attention because it recapitulates certain features of solid tumor tissues, such as cell-ECM interaction and tumor micro-architecture^3^,^4^,^5^,^6^. Many types of 3D scaffolds with different characteristics have been used to culture tumor cells^2^. For example, hydrogels made by natural proteins, such as collagen and Matrigel, have better biological activity, but they have higher batch-to-batch variation^7^. Synthetic polymers, such as PLGA^8^ and PEG^9^, are well-defined and have controllable physical and chemical properties, however, their biological function is lower than natural biomaterials. Therefore, how to improve the biological function and controllability of materials at the same time remains a great challenge. Riching *et al*. prepared 3D collagen gels containing aligned fibers by using a device to impart mechanical strain^10^. They found an aligned matrix could enhance invasion through 3D collagen matrices by matrix topography, rather than stiffness. Loessner *et al*. used biomimetic PEG-based hydrogels to construct a bioengineered 3D ovarian cancer model that could be used as an effective screening tool for the efficacy of therapeutics for intraperitoneal treatment of advanced ovarian cancer^11^. Bray *et al*. constructed 3D *in vitro* bioengineered tumour angiogenesis microenvironments using a glycosaminoglycan-based hydrogel culture system, which could routinely recreate breast and prostate tumour vascularisation^12^. Loessner *et al*. developed gelatine methacrylamide (GelMA)-based hydrogels that combined the biocompatibility of natural materials with the stability and modularity of synthetic materials^13^,^14^.

In addition, our previous work found that alginate hydrogel beads (ALG) increased the metastatic ability of tumor cells and could enrich the cancer stem cell (CSC)-like cells^15^,^16^. Moreover, the CSC-like cell enrichment and the chemoresistance of tumor cells to anticancer drugs could be regulated by the physical properties of ALG^17^,^18^. These results indicate that ALG might offer great potential in tumor microenvironment mimicry. However, bio-inert alginate hydrogel lacks sufficient bioactivity, and thus, modifications are needed to better reproduce the ECM properties of the *in vivo* tumor microenvironment.

Chondroitin sulfate (CS) is a type of sulfated glycosaminoglycan composed of alternating units of β-1,4-linked glucuronic acid (Glca) and β-1,3-N-acetyl-D-glucosamine (GalNAc)^19^. This material is an important ECM component that plays an important role in the ECM structure and in cell functions^20^,^21^, including adhesion, migration, and receptor binding, among others^22^,^23^,^24^. Interestingly, compared with corresponding healthy tissues, the CS expression level is significantly increased within the ECM of many human solid tumors^25^, such as hepatocellular carcinoma (HCC) and head and neck squamous cell carcinoma (HNSCC)^26^,^27^,^28^, which indicates that CS is closely related to tumor occurrence, progression and metastasis^12^,^29^. This abnormal increase of CS expression in the *in vivo* tumor microenvironment inspired us to modify the alginate hydrogel using CS.

In this study, CS-modified alginate hydrogel beads (ALG-CS) were developed to mimic the *in vivo* tumor microenvironment with an abnormally increased expression of CS. We investigated the physical properties, gel formation mechanism and special network structures of ALG-CS. Moreover, we also examined the ability of ALG-CS to promote tumor cell metastasis. We aimed to develop a convenient and effective 3D biomimetic scaffold that could be used to construct standardized tumor metastasis models for tumor research and anticancer drug screening.

## Results

### Physical properties of ALG-CS

**Figure 1:**
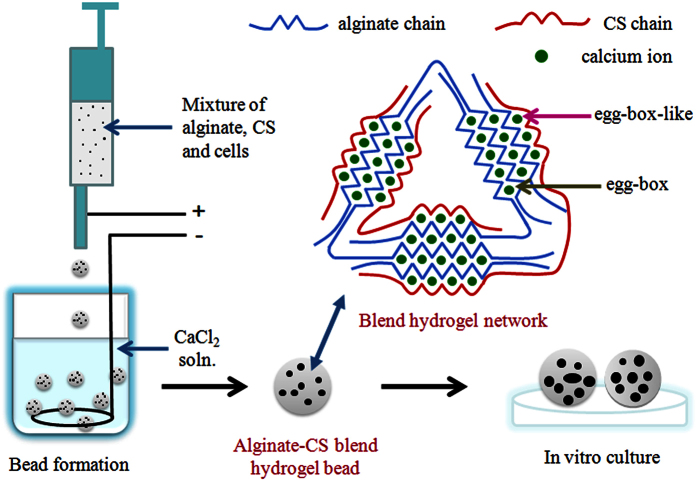
Schematic illustration of the procedure for 3D cell culture in the biomimetic chondroitin sulfate (CS)-modified alginate hydrogel beads (ALG-CS) and the ALG-CS network. Tumor cells were suspended in a mixed solution of alginate and CS. The mixture was extruded into CaCl_2_ solution to form beads using a high-voltage electrostatic droplet generator. Beads containing cells were cultured for 7 days. The traditional alginate hydrogel beads (ALG) without CS were prepared as a control via the same procedure. ALG-CS has a novel network that differs from that of the traditional ALG. Alginate chains not only produce the traditional egg-box structures but also can form asymmetric egg-box-like structures with CS chains via the coordination of calcium ions, which creates a CS-modified biomimetic alginate hydrogel that mimics the tumor microenvironment with increased expression of CS.

**Figure 2:**
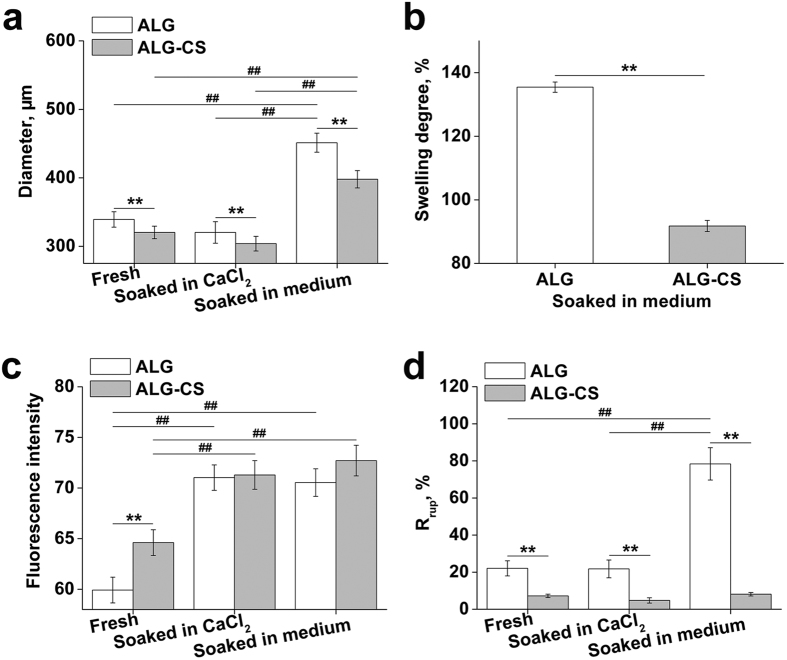
Characterization of size, swelling behavior, permeability and mechanical stability of freshly prepared beads (ALG and ALG-CS), beads soaked in CaCl_2_ solution or beads soaked in cell culture medium for 3 days. (**a**) Diameters of both beads under different conditions. (**b**) Volume swelling degrees of both beads soaked in culture medium. (**c**) Permeability of both beads under different conditions. (**d**) Rupture rate (*R*_*rup*_) of both beads under different conditions. Data are represented as the means ± SD of three independent experiments. **P < 0.01, comparison between ALG and ALG-CS. ^##^P < 0.01, comparison among groups under different conditions.

ALG-CS without cells were generated using the method shown in Fig. 1. The ALG-CS had a spherical morphology that was similar to that of ALG (Fig. S1). The diameter of ALG-CS was significantly smaller than that of ALG for freshly prepared beads (P = 3.93065E-9), beads soaked in CaCl_2_ solution (P = 1.05332E-8) or beads soaked in cell culture medium (P = 0) for 3 days (Fig. 2a). Addition of CS reduced the viscosity of the alginate solution (Fig. S2), which might decrease the diameter of freshly prepared ALG-CS compared with that of ALG. The sizes of both types of beads had a tendency to decrease compared with that of freshly prepared beads when beads were soaked in CaCl_2_ solution, but no significant differences were found (Fig. 2a). However, immersion in cell culture medium resulted in a significant increase in the size of both types of beads compared with that of freshly prepared beads (for ALG P = 0, for ALG-CS P = 0) (Fig. 2a), which might be due to the calcium-sodium ion exchange in the cell culture medium. Additionally, the swelling degree of ALG was significantly higher than that of ALG-CS after soaking in cell culture medium (P = 3.48085E-5) (Fig. 2b).

In addition, freshly prepared ALG-CS had a higher BSA permeability than ALG (P = 1.59662E-4) (Fig. 2c). The permeability of both types of beads was increased significantly after soaking in CaCl_2_ solution or cell culture medium compared with that of freshly prepared beads (for ALG P = 3.32794E-8, for ALG-CS P = 6.18116E-7) (Fig. 2c). However, no significant differences were observed in permeability between beads soaked in CaCl_2_ solution and cell culture medium.

ALG-CS displayed better mechanical stability than ALG under the three conditions, especially in cell culture medium (ALG-CS *R*_*rup*_ 8.1 ± 0.93% vs. ALG *R*_*rup*_ 78.4 ± 8.71%) (P = 1.25371E-7) (Fig. 2d). Immersion in CaCl_2_ solution did not affect the mechanical stability of both types of beads compared with that of freshly prepared beads. However, the *R*_*rup*_ of ALG was significantly increased after soaking in cell culture medium compared with that of freshly prepared ALG (P = 0) (Fig. 2d) probably due to the calcium-sodium ion exchange, whereas the *R*_*rup*_ of ALG-CS did not change significantly.

### Microstructure and elemental composition analysis of ALG-CS

**Figure 3:**
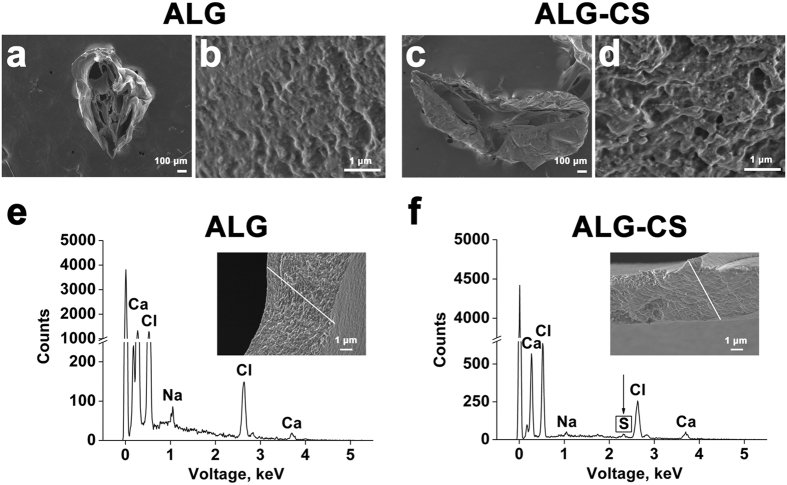
Microarchitecture and qualitative elemental analysis of freshly prepared beads (ALG and ALG-CS) using SEM and EDX detection. (**a,c**) Overall appearance of both beads. Scale bar = 100 μm. (**b,d**) Cross-sectional morphology of both beads. Scale bar = 1 μm. (**e,f**) Individual EDX analysis of both beads. Peaks corresponding to an individual element are marked. The S element was found on the *in situ* cross-section of ALG-CS, which indicated that CS was incorporated within the hybrid hydrogel.

Figure 3 shows the overall appearance and the cross-sectional microstructure of freeze-dried ALG and ALG-CS as observed by SEM. Cavities were found inside the beads (Fig. 3a,c), and pores with larger sizes were found on the cross-section of ALG-CS vs. those on ALG (Fig. 3b,d), which might result in higher permeability of ALG-CS (P = 1.59662E-4) (Fig. 2c). In addition, S element was found only on the *in situ* cross-section of ALG-CS, which indicated that CS was incorporated within the hybrid hydrogel (Fig. 3e,f).

### Content of elemental sulfur and calcium ions in ALG-CS

**Figure 4:**
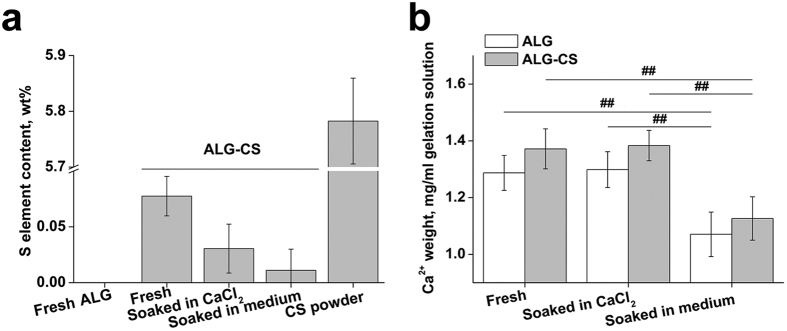
Quantitative assessment of the content of S element and calcium ions incorporated within freshly prepared beads (ALG and ALG-CS), beads soaked in CaCl_2_ solution or beads soaked in cell culture medium for 3 days. (**a**) S element content analysis of both beads under different conditions using an elemental analyzer. CS powder served as the control. (**b**) Weight of calcium ions incorporated within both beads prepared from 1 ml gelation solution (1 ml alginate solution with or without CS) under different conditions detected by ICP-OES. Data are represented as the means ± SD of three independent experiments, ^##^P < 0.01.

The content of elemental sulfur in CS powder was 5.8 ± 0.1 wt% (Fig. 4a). The sulfur content in freshly prepared ALG-CS was 0.08 ± 0.02 wt%, but no elemental sulfur was detected in ALG (Fig. 4a). The sulfur content in ALG-CS had a tendency to decrease gradually after soaking in CaCl_2_ solution or cell culture medium compared with that of freshly prepared beads but without significant difference, which indicated that immersion might drive some CS molecules loss from ALG-CS.

The content of calcium ions in ALG-CS was a slightly higher than that in ALG under the three conditions (Fig. 4b). Immersion in CaCl_2_ solution did not change the content of calcium ions in both types of beads compared with that of freshly prepared beads. However, immersion in cell culture medium significantly decreased the content of calcium ions due to the calcium-sodium ion exchange compared with that of freshly prepared beads (for ALG P = 7.25986E-5, for ALG-CS P = 9.50278E-6) (Fig. 4b).

### Distribution of alginate and CS in ALG-CS

Soaking time in cell culture medium did not affect the distribution of 5-aminofluorescein (AF)-labeled alginate or CS inside both types of ALG-CS, including (AF-labeled-ALG)-CS (Fig. S3b,c) and ALG-(AF-labeled-CS) (Fig. S3f,g). Exact overlap was observed between bright field and fluoroscopic images of (AF-labeled-ALG)-CS (Fig. S4a), which showed that alginate had a uniform and stable distribution. However, for ALG-(AF-labeled-CS), no fluorescence signal was detected on the edge of beads (Fig. S4b, pointed arrow), which indicated that CS located close to the surface of ALG-CS might be more easily lost.

### Isothermal titration calorimetry analysis

**Figure 5:**
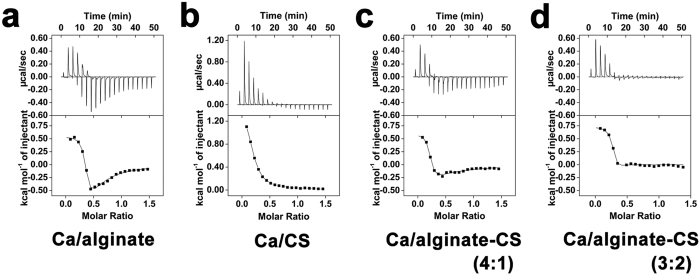
Isothermal titration calorimetry (ITC) thermograms recorded for injection of 10 mM CaCl_2_ into alginate, CS, alginate/CS mixed solutions, and the corresponding binding isotherms according to molar ratio. CaCl_2_ was injected into 2.52 mM alginate solution (Ca/alginate) (**a**), into 0.99 mM CS solution (Ca/CS) (**b**), into the solution containing 2.52 mM alginate and 0.25 mM CS (mass ratio of alginate and CS 4:1) (Ca/alginate-CS 4:1) (**c**), and the solution containing 2.52 mM alginate and 0.66 mM CS (mass ratio of alginate and CS 3:2) (Ca/alginate-CS 3:2) (**d**). Data showed that Ca^2+^ also could bind to CS chains, except for alginate chains.

The isothermal titration calorimetry (ITC) thermogram for alginate showed two-step binding with calcium ions (Fig. 5a). The first step (Step 1) was an endothermic process, and the second (Step 2) was an exothermic process. However, for CS, only one-step binding was observed via an endothermic process (Fig. 5b). Table S1 showed that the binding constant of Step 1 of Ca/alginate (*K*_2_) was much higher than that of Ca/CS. Step 1 of Ca/alginate and Ca/CS was entropy driven, whereas Step 2 of Ca/alginate was enthalpy-entropy driven. The enthalpy and entropy changes of Ca/CS fell between those of Step 1 and Step 2 of Ca/alginate (Table S1).

In addition, when CaCl_2_ was injected into a mixed solution of alginate and CS (mass ratios 4:1), the heat flow versus time profiles (Fig. 5c) were similar to that of alginate alone (Fig. 5a). However, the heat absorption was relatively increased in Step 1, whereas heat release decreased significantly in Step 2 in the mixed solution (Fig. 5c), which might be due to the endothermic reaction between CS and calcium ions. Moreover, when the CS concentration was further increased (mass ratio = 3:2), the exothermic process nearly disappeared (Fig. 5d). These data indicated that calcium ions could be bound to alginate and CS molecules at the same time in the mixed solution, which presents the possibility that ALG-CS is formed by cross-linking between calcium ions and the two types of polymers.

To better understand the gel formation mechanism of ALG-CS, experiments on gel formation and dissolution were also performed. The solution remained clear when CaCl_2_ was added into CS solution (Fig. S5, No. 4), which showed that the coordination between CS and calcium ions was not able to form a gel. However, gels were formed when CaCl_2_ solution was added into alginate solution (Fig. S5, No. 5) or a mixed solution of alginate and CS (Fig. S5, No. 6). Moreover, these gels could be completely dissolved in sodium citrate solution (Fig. S5, No. 7 and No. 8). These phenomena indicated that hybrid hydrogel was also triggered by Ca^2+^. Calcium alginate formed the framework of ALG-CS, and CS molecules could have attached to the gel frame via coordination of calcium ions.

### Quantum chemistry calculation of ALG-CS

The CS used in this study primarily consists of chondroitin-4-sulfate (CSA) and chondroitin-6-sulfate (CSC). For CSA and CSC, sulfation occurs at the 4- and 6-position of the galactosamine moiety, respectively. The optimized structures for an alginate molecule containing two L-guluronic acid residues (GG), CSA and CSC were obtained using the B3LYP method (Fig. S6a,b,c).

**Figure 6:**
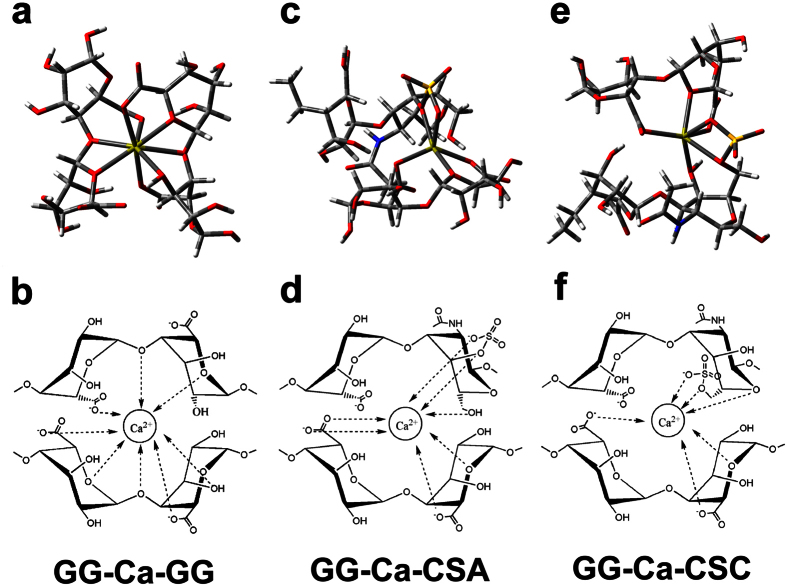
Optimized egg-box and asymmetric egg-box-like structures within hybrid hydrogel via the B3LYP method and the corresponding schematic drawing of calcium coordination of the different structures. (**a,b**) Egg-box structure of alginate-Ca^2+^ formed by two alginate chains composed of two G units and one Ca^2+^ (GG-Ca-GG). (**c–f**) Asymmetric egg-box-like structure of alginate-Ca^2+^-CS formed by one alginate chain composed of two G units, one CSA or CSC chain and one Ca^2+^ (GG-Ca-CSA, GG-Ca-CSC) (red color represents oxygen atom; blue color represents nitrogen atom; white represents hydrogen atom; grey color represents carbon atom; yellow represents sulfur atom; green represents calcium atom). Data showed that the traditional egg-box and asymmetric egg-box-like structures were present in ALG-CS and were triggered by calcium ions.

Two G blocks along the alginate chain formed a buckled region, and Ca^2+^ could be coordinated within this region (GG-Ca) (Fig. S6d). Moreover, a symmetric egg-box structure was formed when Ca^2+^ was coordinated within the cavities created by a pair of the buckled G sequences (GG-Ca-GG) (Fig. 6a,b), as well described in previous studies^30^,^31^. Figure S6e,f show the Ca^2+^ binding structures of CSA and CSC (CSA-Ca and CSC-Ca), respectively. For both CSA and CSC, Ca^2+^ was coordinated to the oxygen atom of the carboxyl group, the oxygen atom on carbon 5 of Glca, the oxygen atom of the sulfonic acid group, and the oxygen atoms on carbon 4 and carbon 6 of GalNAc. However, CSA-Ca and CSC-Ca had distinct conformations due to the different positions of sulfation on GalNAc.

Table S2 showed that the chemical bond of GG-Ca (bond energy 463.59 kcal/mol) was significantly stronger than that of CSA-Ca (338.91 kcal/mol) or CSC-Ca (350.20 kcal/mol). The chemical bond of CSC-Ca was relatively stronger than that of CSA-Ca, which showed that sulfation at the 6-position of GalNAc might be better for Ca^2+^ binding than at the 4-position.

Moreover, Ca^2+^ also could be coordinated within the cavities created by GG and CSA or CSC, which generated novel asymmetric egg-box-like structures, GG-Ca-CSA (Fig. 6c,d) and GG-Ca-CSC (Fig. 6e,f). The Ca^2+^ was located closer to the sulfate ester group in GG-Ca-CSA or GG-Ca-CSC, whereas the GG-Ca-GG structure was compact and symmetric (Fig. 6a,b). These data indicated that the modification mechanism of CS on alginate hydrogel was due to the cross-linking between CS and alginate molecules via coordination of calcium ions, which formed novel asymmetric egg-box-like structures in hybrid hydrogel.

Similar to GG-Ca, CSC-Ca and CSA-Ca, the chemical bond of GG-Ca-GG was strongest and that of GG-Ca-CSA was weakest (Table S2). The sulfation at the 6-position of GalNAc also enhanced the Ca^2+^ binding of GG-Ca-CS compared with sulfonation at the 4-position.

### Live/dead and viability of tumor cells in ALG-CS

**Figure 7:**
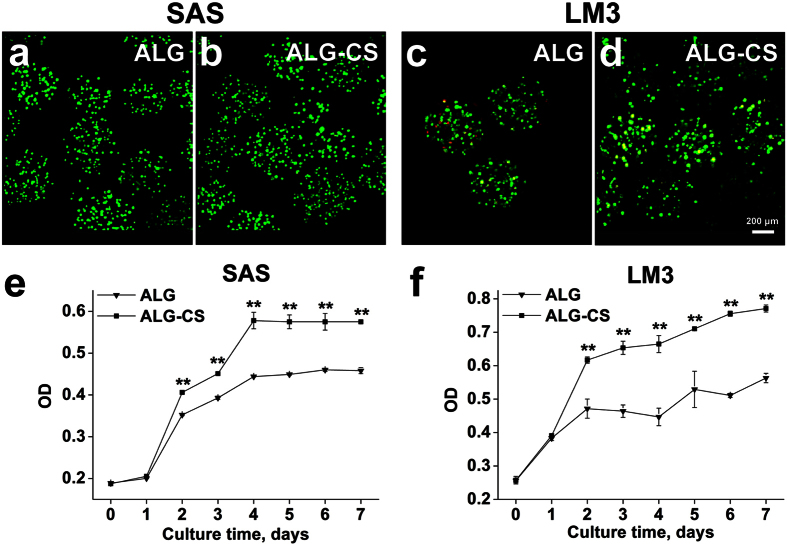
Higher viability and growth of tumor cells cultured in ALG-CS compared with in ALG. Live/dead staining of SAS cells (**a,b**) and LM3 cells (**c,d**) cultured in both beads were performed on Day 7. Scale bar = 200 μm. Cell viability of SAS (**e**) and LM3 cells (**f**) were detected using CCK-8 assay. Data are represented as the means ± SD of three independent experiments, **P < 0.01.

Both the human HNSCC cell line SAS and the human HCC cell line HCCLM3 (LM3) were still alive in ALG-CS after 7 days of culture (Fig. 7b,d), and no significant differences were noted in tumor cell morphology between ALG and ALG-CS (Fig. 7a–d). However, the viability of both SAS and LM3 cells was significantly higher in ALG-CS compared with that in ALG since Day 2 (for SAS cells P = 2.29013E-6 on Day 2, P = 5.90075E-5 on Day 3, P = 3.04886E-4 on Day 4, P = 1.92181E-4 on Day 5, P = 5.4408E-4 on Day 6, P = 1.75869E-5 on Day 7) (for LM3 cells P = 0 on Day 2, P = 2.63972E-4 on Day 3, P = 4.88895E-4 on Day 4, P = 0 on Day 5, P = 1.77364E-6 on Day 6, P = 1.77364E-6 on Day 7) (Fig. 7e,f), which indicated that the addition of CS was beneficial to enhancing tumor cell viability.

### Metastatic properties of tumor cells in ALG-CS

**Figure 8:**
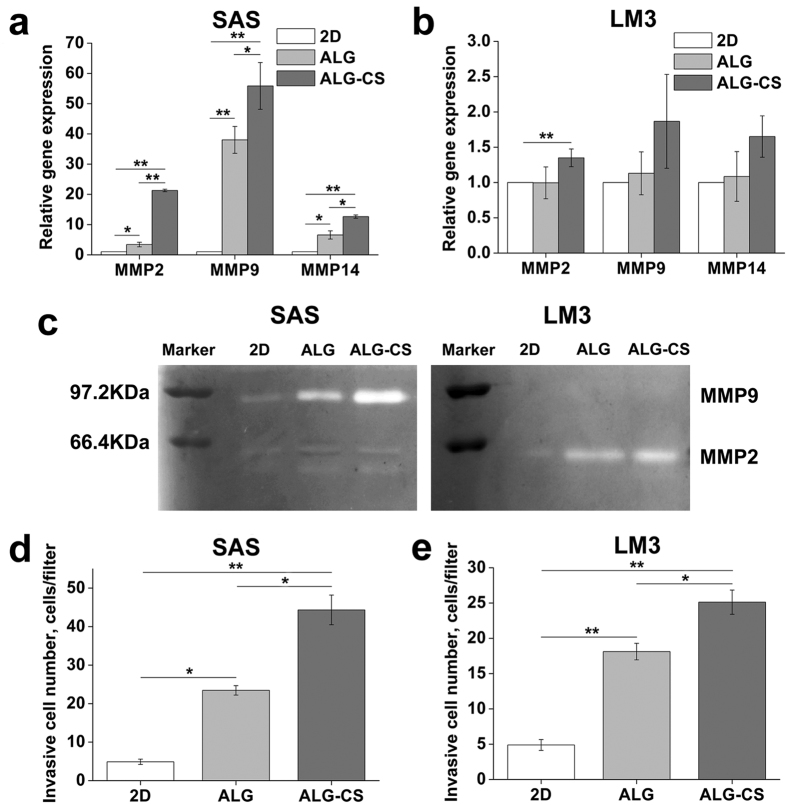
Higher metastatic potential of tumor cells cultured in ALG-CS on Day 7 compared with in ALG. (**a,b**) Relative gene expression of MMPs in SAS and LM3 cells cultured in both beads as detected by quantitative real-time PCR. (**c**) Secreted MMP2 and MMP9 evaluated by zymography. (**d,e**) The cell number invaded through Matrigel-coated filter membrane was statistically analyzed. Adhesion cells cultured in T-flasks served as the controls (2D). Data are represented as the means ± SD of three independent experiments. *P < 0.05 and **P < 0.01.

It was found that the gene expression of matrix metalloproteinase (MMP) (MMP2, MMP9 and MMP14) was highest in both SAS and LM3 cells cultured in ALG-CS, while lowest in 2D, especially for SAS cells (Fig. 8a,b). For SAS cells, the gene expression of MMP2, MMP9 and MMP14 in ALG-CS was significantly higher than that in ALG (for MMP2 P = 0, for MMP9 P = 0.04634, for MMP14 P = 0.00194), which indicated that ALG-CS could further enhance MMP gene expression in tumor cells compared with ALG.

For SAS cells, the zymograms and semi-quantitative analysis showed MMP9 activity was significantly higher than that in 2D (P = 1.86861E-4) (Fig. 8c and Fig. S7a). Moreover, MMP9 activity was significantly further enhanced in ALG-CS compared with that in ALG (P = 1.39247E-4) (Fig. 8c and Fig. S7a). However, low MMP2 activity was detected in all groups without significant differences (Fig. 8c). For LM3 cells, MMP2 activity was significantly higher in ALG or ALG-CS compared with that in 2D (for ALG P = 0.00215, for ALG-CS P = 7.89685E-4) (Fig. 8c and Fig. S7b). And MMP2 activity was relatively higher in ALG-CS than that in ALG without significant differences (Fig. 8c and Fig. S7b). However, MMP9 secretion was undetected in LM3 cells in all groups (Fig. 8c). These data indicated that the specific MMP activity could be further increased in ALG-CS compared with that in ALG.

Invasive activity has a close relationship with metastatic potential. For both cell lines, the migration of cells through Matrigel-coated membranes was increased significantly in ALG compared with that in 2D (for SAS cells P = 0.0017, for LM3 cells P = 9.51353E-6) (Fig. 8d,e), as reported in our previous study^7^. Moreover, for SAS and LM3 cells, tumor cell invasiveness was further increased by 5-fold and 1.4-fold in ALG-CS compared with that in ALG, respectively (for SAS cells P = 0.00218, for LM3 cells P = 0.00469) (Fig. 8d,e), which indicated that tumor cell metastasis could be significantly enhanced in ALG-CS.

## Discussion

It is known that a 2D tumor model poorly predicts clinical outcomes in patients with malignant tumors due to the lack of *in vivo*-like ECM and tissue structures^2^,^5^,^6^,^31^. Animal models also present important shortcomings such as cost and time, and they are not helpful in uncovering the molecular mechanisms at the early stage of diseases^5^,^31^. Tissue-engineered humarized xenograft models are more bio-similar to real life scenarios, which helps us to understand the interaction between human tumor cells and the specific humanized tissue microenvironment^32^,^33^. However, *in vitro* standardized 3D tumor models also should be developed for high throughput drug screening considering the complexity and time-consuming of *in vivo* models.

Many types of naturally occurring materials or synthetic polymers are used in tumor cell 3D culture^2^. Among these, alginate is a naturally occurring anionic polysaccharide with a structure similar to that of glycosaminoglycans in the *in vivo* ECM^2^. The gelation and dissolution of alginate gel occurs under physiological conditions without any toxicity, which is better for cell viability and easier for subsequent analysis^2^,^17^. And ALG have a good degree of sphericity with controllable diameters and can be generated at a large scale under standardized conditions^15^,^18^. For example, we have built up a mathematical model to generate ALG with desired matrix stiffness^18^. Moreover, our previous studies showed that ALG could promote CSC-like cell proportion enrichment and increase the metastatic activity, tumorigenicity and drug resistance of tumor cells, because ALG could recapitulate certain features of *in vivo* tumor microenvironment to some extent, such as hypoxia and matrix stiffness^15^,^16^,^17^,^18^. However, large differences still exist between ALG and the ECM of the *in vivo* tumor tissues due to the bio-inertness of alginate. Therefore, in this study, ALG-CS was developed to mimic the *in vivo* tumor microenvironment with abnormally increased expression of CS^25^.

We investigated the mechanism of ALG-CS gel formation. It was found that calcium ions could be bound to alginate and CS molecules at the same time (Fig. 5c,d), which formed novel asymmetric egg-box-like structures, including GG-Ca-CSA (Fig. 6c,d) and GG-Ca-CSC (Fig. 6e,f), except the traditional egg-box structure GG-Ca-GG (Fig. 6a,b). And the traditional egg-box structure contributed to the formation of the gel framework of ALG-CS, whereas the asymmetric egg-box-like structure allowed CS to attach to the gel frame (Fig. S5), which generated the hybrid hydrogel network (Fig. 1). Although the calcium-sodium ion exchange resulted in the loss of some CS molecules attached to alginate chains by Ca^2+^ coordination during the immersion in cell culture medium (Fig. 4a), many CS molecules still existed in ALG-CS after 7-day immersion (Fig. S3g).

We also evaluated the mechanical stability of ALG and ALG-CS soaked in CaCl_2_ solution or cell culture medium for 3 days (Fig. 2d). When both kinds of beads were soaked in CaCl_2_ solution, we found the mechanical stability of them did not change compared with that of freshly prepared beads (Fig. 2d), which was due to the stable content of calcium ions in both types of beads (Fig. 4b). However, when ALG was soaked in cell culture medium for 3 days, its mechanical stability was significantly decreased compared with freshly prepared beads (Fig. 2d), which was due to the loss of some calcium ions (Fig. 4b). Although ALG-CS also lost calcium ions like ALG, the addition of CS could retain relatively more calcium ions compared with ALG (Fig. 4b), which could cross-link alginate and CS molecules to form asymmetric egg-box-like structures by the coordination reaction. So ALG-CS had better mechanical stability than ALG when soaked in cell culture medium for 3 days. We also investigated the mechanical stability of both kinds of beads soaked in cell culture medium for 7 days. It was found that the *R*_*rup*_ of ALG-CS was also increased to 100% like ALG in medium on Day 7 (Fig. S8), which indicated that the continuous loss of calcium ions in medium would damage asymmetric egg-box-like structures in ALG-CS. So the positive effect of CS on the mechanical stability of beads significantly declined at the late stage of immersion in medium (between Day 3–7).

In addition, egg-box-like structures, GG-Ca-CSA and GG-Ca-CSC (Fig. 6), were asymmetric, and their bond energies were lower than GG-Ca-GG (Table S2), which might increase the interspacing of the gel network and the BSA permeability (Fig. 2c) compared with those in ALG.

In this study, we found that the cell viability of the two cell lines was increased significantly in ALG-CS compared with in ALG (Fig. 7e,f). Two factors might be involved in this result. First, CS might increase mRNA and DNA biosynthesis and promote cell metabolism^34^. Second, ALG-CS had better permeability than ALG (Fig. 2c), which could improve the efficiency of mass transfer.

It has been reported that the expression of CS was increased significantly in the ECM of many tumor tissues, which mediates cell adhesion and facilitates tumor cell invasion^24^,^35^. In this study, ALG-CS could mimic the *in vivo* tumor microenvironment with abnormally increased expression of CS to a certain degree, which significantly enhanced the metastasis of tumor cells compared with ALG. MMPs are known as important molecules that aid tumor cells during metastasis^36^. It is well accepted that enhanced expression of MMPs increases tumor cell invasion^37^. Our data showed that SAS and LM3 cells cultured in ALG-CS had higher gene expression of MMP2, MMP9 and MMP14 than cells in ALG (Fig. 8a,b). For SAS cells, the secretion of active MMP9 in ALG-CS was significantly increased compared with in ALG (Fig. 8c and Fig. S7a), which was consistent with the MMP9 gene expression. And the secretion of active MMP2 for SAS cells was quite low in all three groups (Fig. 8c), as reported in a previous study^38^. In contrast to SAS cells, LM3 cells significantly increased the secretion of active MMP2 in ALG-CS compared with in ALG (Fig. 8c and Fig. S7b), consistent with the MMP2 gene expression. The secretion of active MMP9 for LM3 cells was too low to detect in all three groups (Fig. 8c), consistent with our previous study^15^. These data indicated that the specific MMP activity could be further increased in ALG-CS and was also dependent on different cell types. In this study, we used RT-PCR and zymography techniques to reflect the expression of MMP2 and MMP9 on mRNA and protein level. The difference between gene expression and proteinases activity might be caused by different analytical sensitivities. Similar results have been reported by other group^39^. Moreover, the *in vitro* invasion assay further confirmed that the migration of both SAS and LM3 cells through Matrigel-coated membranes was increased significantly in ALG-CS compared with that in ALG (Fig. 8d,e), which might be related to the upregulated secretion of active MMP9 and MMP2, respectively. Although it was found that the activity of MMPs and cell invasion ability were enhanced in ALG-CS in this study. However, it is necessary to evaluate the alterations in cell surface and secreted macromolecules, such as proteoglycans, which will help to elucidate mechanisms in tumor metastasis and treat cancer^40^.

TIMP1 and TIMP2 are the tissue inhibitors of MMP9 and MMP2, respectively. In this study, we found that the TIMP1 gene expression in SAS cells cultured in ALG was significantly lower than that in 2D cells and there was no significant difference in TIMP1 gene expression between ALG and ALG-CS (Fig. S9a), which was consistent with the report on TIMP1 in oral squamous cell carcinoma tissues^41^. In addition, we found that higher expression of TIMP1 gene in the 3D-cultured LM3 cells (Fig. S9b). Higher gene expression of TIMP1 was detected in hepatocellular carcinoma compared with normal uninvolved liver and the tumor margin^42^. No significant difference in TIMP2 gene expression was detected in SAS and LM3 cells (Fig. S9a,b).

Invadopodia formation plays an important role during tumor invasion^43^. The invadopodia degrades ECM through the presence of matrix degrading enzymes, such as MMPs^44^. In the decades, with constant understanding of new roles of MMPs in cancer, it might provide new opportunities for the design of pharmacological targeting of MMPs^45^. Several approaches, such as innovations in chemical design, antibody-based strategies, and nanotechnologies, have been used to design MMP inhibitors that target and measure the activity of MMPs^46^.

**Figure 9:**
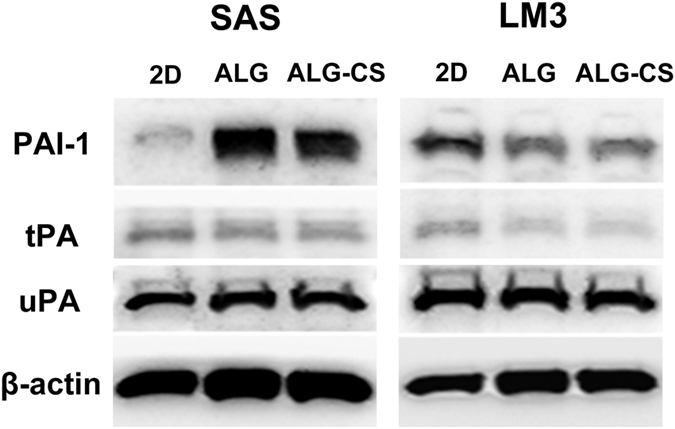
Protein expression of PAI-1, tPA and uPA in SAS and LM3 cells cultured in ALG and ALG-CS as detected by western blot.

Several studies have demonstrated the implication of the uPA in MMPs activation^47^. The uPA system is a key player in the break-down of extracellular matrix and basement membrane that leads to metastasis^48^. Elevated expression of uPA is observed in numerous cancer types and associated with poor prognosis^49^. In this study, we investigated the protein expression of uPA, tPA and PAI-1 in SAS and LM3 cells cultured in ALG and ALG-CS. It was found that the uPA expression was significantly higher than the tPA expression in both cell lines in all groups (Fig. 9). No significant differences in uPA or tPA expression were found in each cell line in different groups (Fig. 9). PAI-1 is a major inhibitor of uPA system and its expression is considered as a poor prognostic indicator in patients with cancer^50^. Higher expression of PAI-1 in HNSCC is considered to be related with poor prognosis in patients^51^,^52^. We found that the PAI-1 expression in SAS cells was significantly increased in either ALG and ALG-CS groups compared to 2D (Fig. 9), which indicated that SAS cells cultured in 3D culture system based alginate might have higher metastatic ability. Previous study shows that the cell invasion is significantly increased after PAI-1 konckdown whereas there is a significant decrease for invasiveness with PAI-1 overexpression^53^. In this study, the PAI-1 expression in LM3 cells cultured in either ALG or ALG-CS was relatively lower than that in 2D cells (Fig. 9), which might be related with higher metasitasic ability detected in 3D cultured cells (Fig. 8).

## Conclusion

In this study, we developed ALG-CS to mimic the *in vivo* tumor microenvironment with an abnormally increased expression of CS for the promotion of tumor cell metastasis. We confirmed that the modification mechanism of CS on alginate hydrogel was due to the cross-linking between CS and alginate molecules via coordination of calcium ions, which formed novel asymmetric egg-box-like structures within ALG-CS and enabled ALG-CS to possess significantly different physical properties compared with ALG. Moreover, we found that both viability and metastasis of tumor cells were significantly enhanced in ALG-CS compared with ALG. The presented findings, herein, showed that ALG-CS could be a convenient and effective 3D biomimetic scaffold that would be used to construct standardized tumor metastasis models for tumor research and anticancer drug screening.

## Methods

### Materials and cell lines

All chemicals were purchased from Sigma-Aldrich (St. Louis, MO, USA) unless otherwise specified. Sodium alginate (molecular weight 500 kDa, G:M ratio 33:67) was purchased from Qingdao Jingyan Bio-Tech Co., Ltd (Qingdao, Shandong, China). The human HNSCC cell line SAS was kindly provided by National Tokyo Medical Center (Tokyo, Japan). The human HCC cell line HCCLM3 (LM3) was obtained from Zhongshan Hospital, Fudan University (Shanghai, China). Dulbecco’s Modified Eagle’s Medium (DMEM) was purchased from Invitrogen (San Diego, CA, USA), and fetal bovine serum (FBS) was purchased from Thermo Scientific (Pittsburgh, PA, USA).

### Preparation of ALG-CS

Sodium alginate or CS sodium was dissolved in saline to form final concentrations of 2% or 5% (w/v), respectively. The two types of solutions were mixed together with a mass ratio of sodium alginate to CS sodium of 4:1. The mixture was extruded into a 100 mM calcium chloride solution from a syringe equipped with a 25-gauge needle and dropped at a voltage of 5 kV. The gelation time necessary to produce ALG-CS was 30 min. The traditional ALG was prepared as a control via the same procedure. Freshly prepared beads (ALG-CS and ALG), beads soaked in CaCl_2_ solution or beads soaked in cell culture medium for 3 days were used for further analysis. The morphology of the beads was observed using an inverted phase-contrast microscope (Eclipse, Nikon, Tokyo, Japan). The diameters of the beads under different conditions were measured using Image J software (the U.S. National Institutes of Health) and the microscope images. For each group, 50 randomly selected beads were analyzed.

### Swelling degree of ALG-CS

The swelling degrees of beads were determined as previously described^54^. The swelling degree was calculated using Equation (1), where *D*_*0*_ is the mean diameter of freshly prepared beads, and *D*_*s*_ is the mean diameter of beads soaked for 3 days.


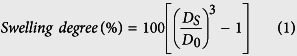


### Permeability of ALG-CS

The permeability of beads was determined as previously described^55^.

### Mechanical stability of ALG-CS

The mechanical stability of beads was determined according to a previously reported method^54^. The rupture rate (*R*_*rup*_) was calculated using Equation (2), where *N* is the total number of beads, and *N*_*r*_ is the number of ruptured beads.


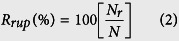


### Microstructure analysis and energy-dispersive X-ray spectroscopy

The microstructures of beads were observed using SEM (JSM-7800F, JEOL, Akishima, Tokyo, Japan). The SEM was coupled to an X-ray detector (X-Max, Oxford Instruments, Abingdon, Oxfordshire, UK). Higher voltage (5 kV) was coupled to the X-ray detector for element mapping of the cross-sections of the beads.

### Analysis of the sulfur content in ALG-CS

Beads were freeze-dried and the sulfur content was determined by Elmentar Vario MICRO cube (Elementar Analysensystene, GmbH, Germany). The CS powder served as the control.

### Amount of calcium ions in ALG-CS

10 ml gelation solution (alginate solution with or without CS) was used to prepare beads. After washing with ddH2O, the beads were dissolved in 55 mM sodium citrate. And the calcium ion concentration was determined using an inductively coupled plasma optical emission spectrometer (ICP-OES) (Optima 7300DV, Perkin-Elmer, Shelton, CT, USA). Data were expressed as the calcium ion weight in the beads prepared from 1 ml of gelation solution.

### Distribution of alginate and CS in ALG-CS

Alginate and CS were fluorescence labeled with 5-aminofluorescein (AF) as previously described^56^. Two types of ALG-CS, including (AF-labeled-ALG)-CS and ALG-(AF-labeled-CS), were prepared using AF-labeled alginate and unlabeled CS, and unlabeled alginate and AF-labeled CS, respectively. The distributions of AF-labeled polymers in freshly prepared beads and beads soaked in culture medium were observed by a confocal laser scanning microscope (CLSM, Leica SP2, Leica Microsystems, Germany).

### Isothermal titration calorimetry

A high-sensitivity isothermal titration calorimeter (MicroCal iTC200, Malvern Instruments Ltd, Worcestershire, UK) was used to investigate the binding of calcium ions to alginate, CS or their mixture. Aliquots (2 μl) of 10 mM CaCl_2_ were continuously injected into a 200 μl calorimeter cell containing 2.52 mM alginate in saline, 0.99 mM CS in saline or 2.52 mM alginate solution supplemented with CS (alginate/CS mass ratios of 4:1 or 3:2, CS 0.25 mM or 0.66 mM). A blank experiment in which CaCl_2_ was titrated into saline was also used to subtract the heat of dilution of CaCl_2_. The binding constant (*K*) and the molar binding enthalpy (Δ*H*) were analyzed using the software provided by the manufacturer. The Gibbs free energy (Δ*G*) and the molar binding entropy (Δ*S*) were calculated.

### Quantum chemistry calculation

Density functional theory (DFT) calculations were performed using the B3LYP^57^, PBEPBE^58^ and M06-2x^59^ methods in conjunction with the 6-31G basis set. All structures were fully optimized in the gas phase, and the vibrational frequencies were calculated at the same level of theory to characterize the nature of the stationary points. All computations were performed with the GAUSSIAN-09 program package (Gaussian, Inc., Wallingford CT, 2009.).

### Cell culture and encapsulation

The SAS and LM3 cells were maintained in DMEM containing 10% (v/v) FBS. Cells were subcultured at approximately 90% confluence. A schematic illustration of the method used to generate ALG-CS containing cells is shown in Fig. 1. In brief, single cells were suspended in 2% (w/v) sodium alginate containing CS (mass ratio of sodium alginate to CS = 4:1) at a cell density of 10^6^ cells/ml. ALG-CS containing cells were prepared and cultured for 7 days. The traditional ALG were prepared as a control. After culture, cells were harvested from both types of beads by treatment with 55 mM sodium citrate and used for gene expression analysis and *in vitro* invasion assay.

### Live/dead assay

Live/dead staining was performed according to the method reported in our previous study^17^.

### Cell viability assay

Cell viability was detected according to the method reported in our previous study^17^.

### Quantitative real-time reverse transcription-polymerase chain reaction (Quantitative real-time PCR)

Quantitative real-time PCR was performed according to the method reported in our previous study^17^. The primers used in this study were listed in Table S3.

### Zymography

Matrix metalloproteinase (MMP) 2 and MMP9 enzymatic activity was determined according to the method reported in previous studies^17^. The zymograms were also scanned, and semi-quantitative analysis was performed using Image J software.

### *In vitro* invasion assay

The invasiveness of 3D cultured cells and monolayer-cultured cells (2D) was evaluated via trans-well assay according to the method reported in our previous study^15^.

### Western blotting

Immunoblotting was carried out using standard techniques. Briefly, cells were lysed in ice-cold 1x RIPA lysis buffer and protein concentrations were determined. Aliquots (50 μg) of protein were denatured in Laemmli loading buffer and separated on precast 4–10% NuPAGE Novex 4–12% Bis-Tris Protein Gels (Life technologies, Carlsbad, CA). Proteins were transferred to polyvinylidene difluoride membranes, which were blocked and probed with primary antibody (PAI-1, tPA, uPA, β-actin, Proteintech Technology, Inc., Wuhan, China), and then detected using appropriate horseradish peroxidase (HRP)-labeled secondary antibodies. Proteins were visualized using enhanced chemiluminescence (Pierce, Thermo-Fisher) on Hyperfilm (GE Healthcare).

### Statistical analysis

All individual experiments were performed in triplicate. Data were presented as the means ± standard deviation (SD). Student’s t-test was applied to detect significant differences for comparison of two groups. One-way analysis of variance (ANOVA) was performed for multiple comparisons. Differences were considered significant for P < 0.05 (* and ^#^) and especially significant for P < 0.001 (** and ^##^).

## Additional Information

**How to cite this article**: Liu, Y. *et al*. Development of a Biomimetic Chondroitin Sulfate-modified Hydrogel to Enhance the Metastasis of Tumor Cells. *Sci. Rep.* **6**, 29858; doi: 10.1038/srep29858 (2016).

## Supplementary Material

Supplementary Information
