# Supplementary material for: Development of a Biomimetic Chondroitin Sulfate-modified Hydrogel to Enhance the Metastasis of Tumor Cells
Source: Sci Rep. 2016 Jul 19;6:29858. doi: 10.1038/srep29858 (PMC4949442; doi:10.1038/srep29858)
Supplement: Supplementary Information [file srep29858-s1.doc]

**Supplementary Table S1-S3 and Fig. S1-S9**

**Development of a Biomimetic Chondroitin Sulfate-modified Hydrogel to Enhance the Metastasis of Tumor Cells**

Yang Liu^1^, Shujun Wang^2,3^, Dongsheng Sun^1,3^, Yongdong Liu^4^, Yang Liu^1^, Yang Wang^1,5^, Chang Liu^1,6^, Hao Wu^1,3^, Yan Lv^2,3^, Ying Ren^2,3^, Xin Guo^2^, Guangwei Sun^1, *^, Xiaojun Ma^2^

^1^ Scientific Research Center for Translational Medicine, Department of Biotechnology, Dalian Institute of Chemical Physics, Chinese Academy of Sciences, Dalian, 116023, China

^2^ Department of Biotechnology, Dalian Institute of Chemical Physics, Chinese Academy of Sciences, Dalian, 116023, China

^3^ University of Chinese Academy of Sciences, Beijing 100049, China

^4^ College of Life Science & Bioengineering, Beijing University of Technology, Beijing, 100124, China

^5^ School of Life Science, Dalian University, Dalian, 116023, China

^6^ Dalian Municipal Central Hospital, Dalian, 116033, China

*Corresponding author. Scientific Research Center for Translational Medicine, Department of Biotechnology, Dalian Institute of Chemical Physics, Chinese Academy of Sciences, 457 Zhongshan Road, Dalian 116023, PR China. Tel. /fax: +86-411-82463027.

E-mail address: sungw@dicp.ac.cn (Guangwei Sun)


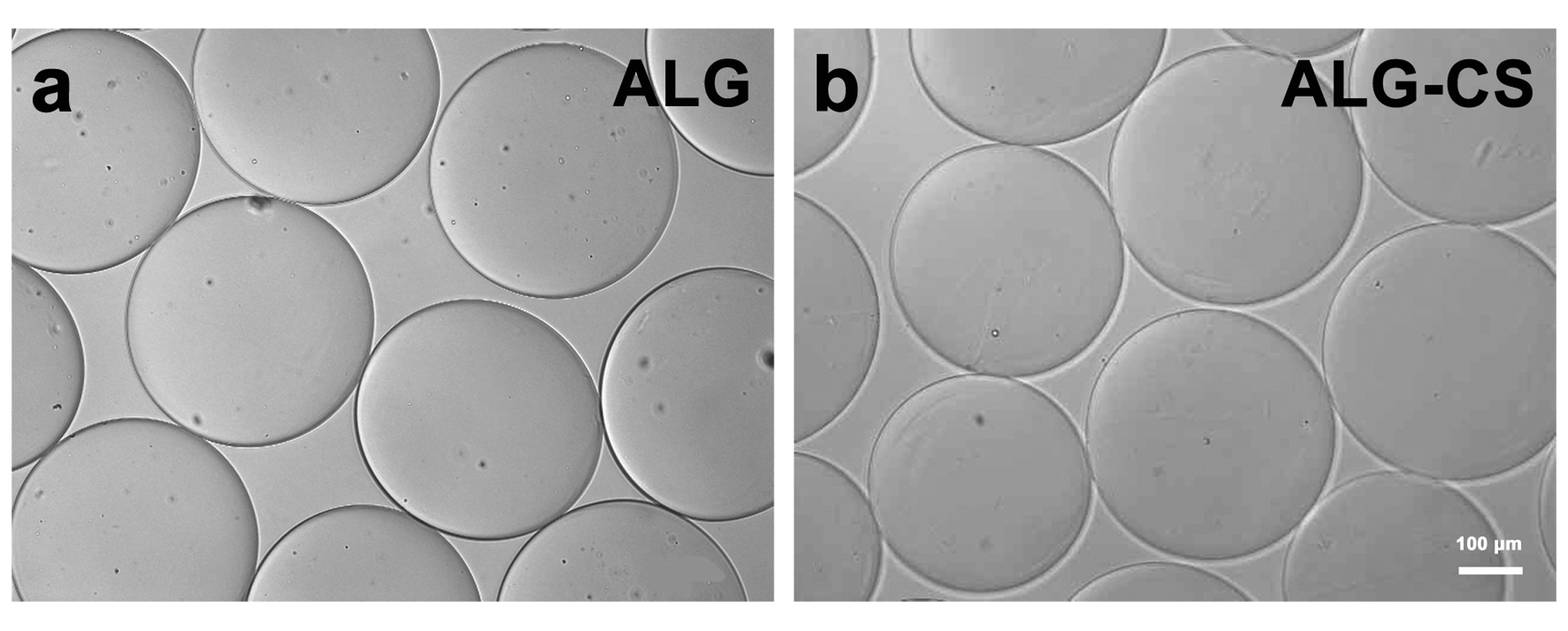


**Figure S1. Similar morphology of freshly prepared ALG (a) and ALG-CS (b).**


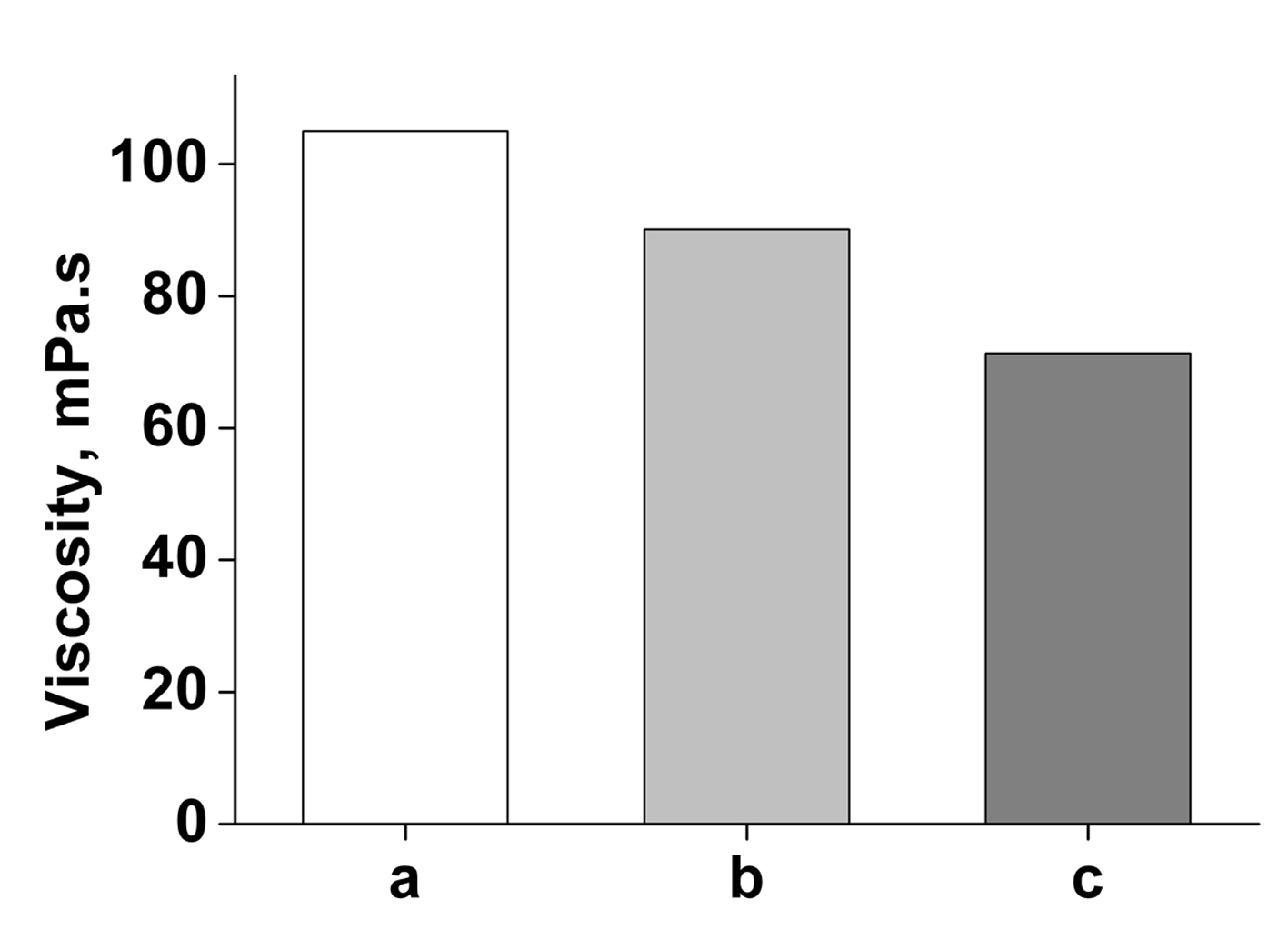


**Figure S2. Viscosity of alginate solutions with or without CS.**

a---2% (w/v) alginate solution;

b---Mixture of 2% (w/v) alginate solution and 5% (w/v) CS solution (mass ratio of alginate and CS 4:1);

c---Mixture of 2% (w/v) alginate solution and physiological saline. The volume of physiological saline was the same as the above 5% (w/v) CS solution.


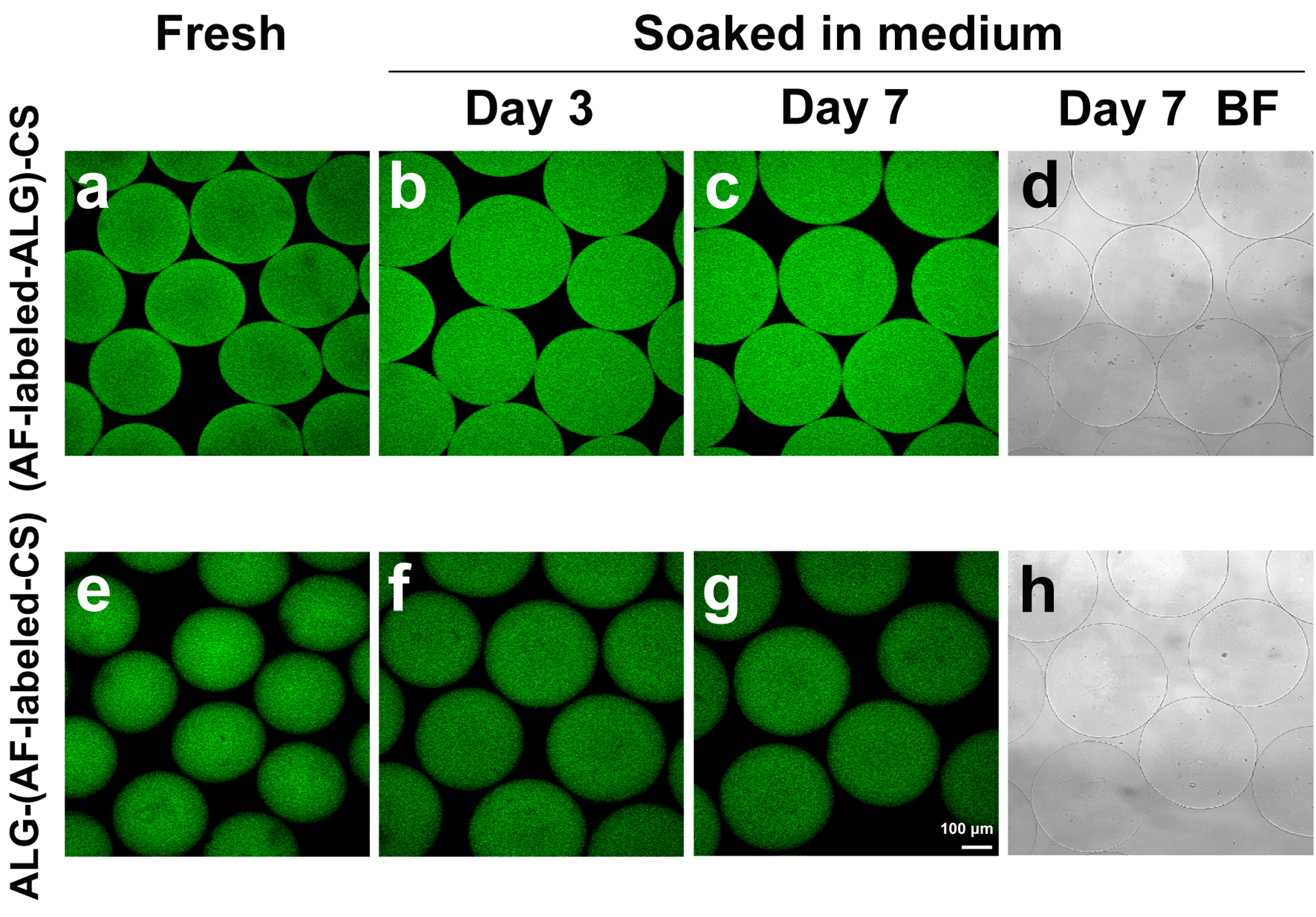


**Figure S3. Confocal laser scanning microscope (CLSM) images of (AF-labeled-ALG)-CS and ALG-(AF-labeled-CS) under different conditions.** (**a, e**) Freshly prepared beads. (**b, c, f, g**) Beads soaked in cell culture medium for 3 and 7 days, respectively. (**d, h**) Corresponding bright field images of beads soaked in medium for 7 days. These fluorescence-labeled beads were observed under the same experimental conditions. Scale bar=100 μm. Data showed that even after soaking in medium for 7 days, an abundance of CS molecules were still incorporated within ALG-CS.


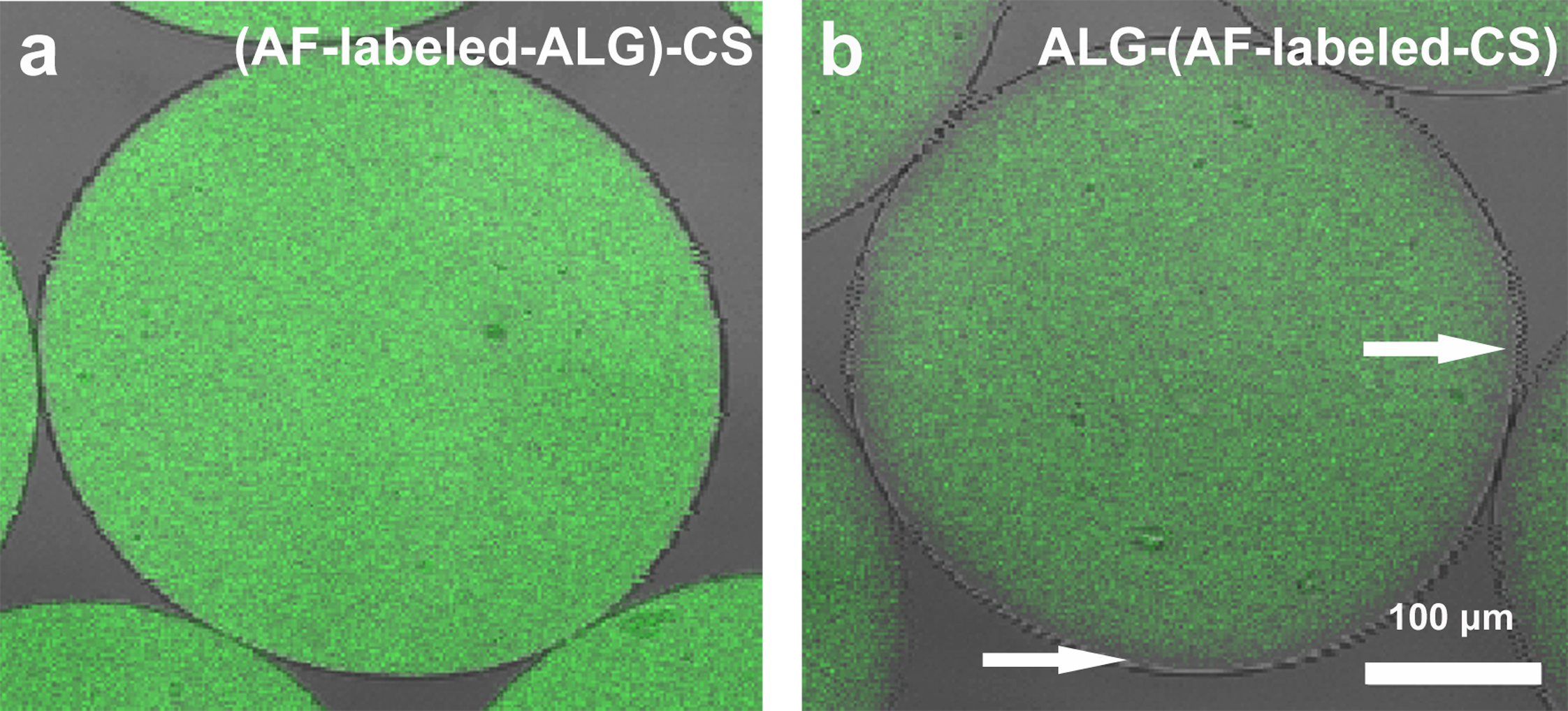


**Figure S4. Distribution of alginate (a) and CS (b) within ALG-CS.** Alginate and CS were fluorescence labeled with 5-aminofluorescein (AF) prior to preparation of beads. Arrow showed that no fluorescence signal was detected on the edge of ALG-(AF-labeled-CS).

**Table S1.** Binding parameters obtained from curve fitting of the binding isotherms for Ca/alginate and Ca/CS.

| **Sample** |  | ***K* (10^3^/M)** | **△*H* (kJ/mol)** | **△*G* (kJ/mol)** | **△*S* (J mol^-1^ K^-1^)** |
| --- | --- | --- | --- | --- | --- |
| **Ca/alginate** | Step 1 | *K*_1_ 2.13 | △*H_1_* 11.7 | △*G*_1_ -19.01 | △*S*_1_ 24.53 |
|  | Step 2 | *K*_2_ 191 | △*H*_2_ -2.29 | △*G*_2_ -34.67 | △*S*_2_ 108.6 |
| **Ca/CS** |  | 8.05 | 9.01 | -22.32 | 105.09 |


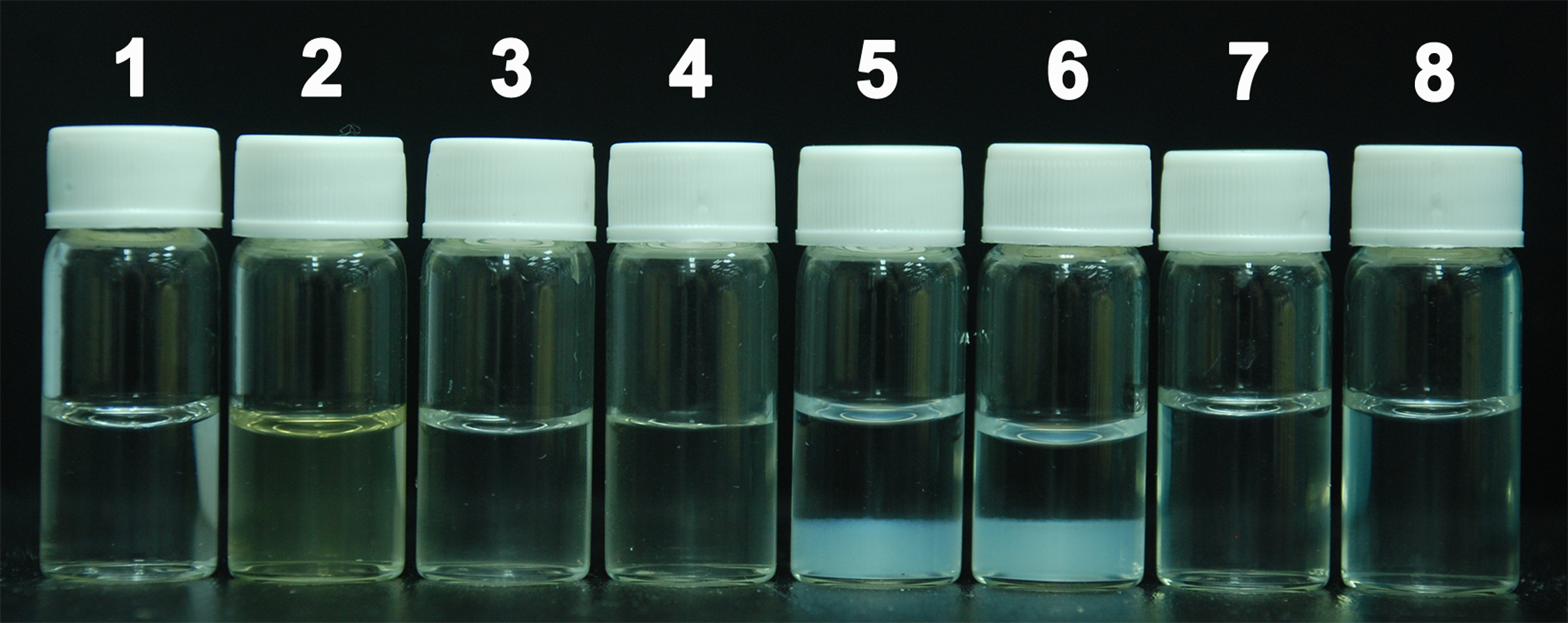


**Figure S5. Photograph of several types of solutions, ALG and ALG-CS.**

No.1---2% (w/v) alginate solution;

No.2---5% (w/v) CS solution;

No.3---Mixture of 2% (w/v) alginate solution and 5% (w/v) CS solution (mass ratio of alginate and CS 4:1);

No.4---Mixture of 5% (w/v) CS solution and excess 0.11% (w/v) CaCl_2_ solution;

No.5---ALG prepared from 2% (w/v) alginate solution;

No.6---ALG-CS prepared from the mixture of 2% (w/v) alginate solution and 5% (w/v) CS solution (mass ratio of alginate and CS 4:1);

No.7---ALG dissolved in excess 55 mM sodium citrate solution.

No.8---ALG-CS dissolved in excess 55 mM sodium citrate solution;


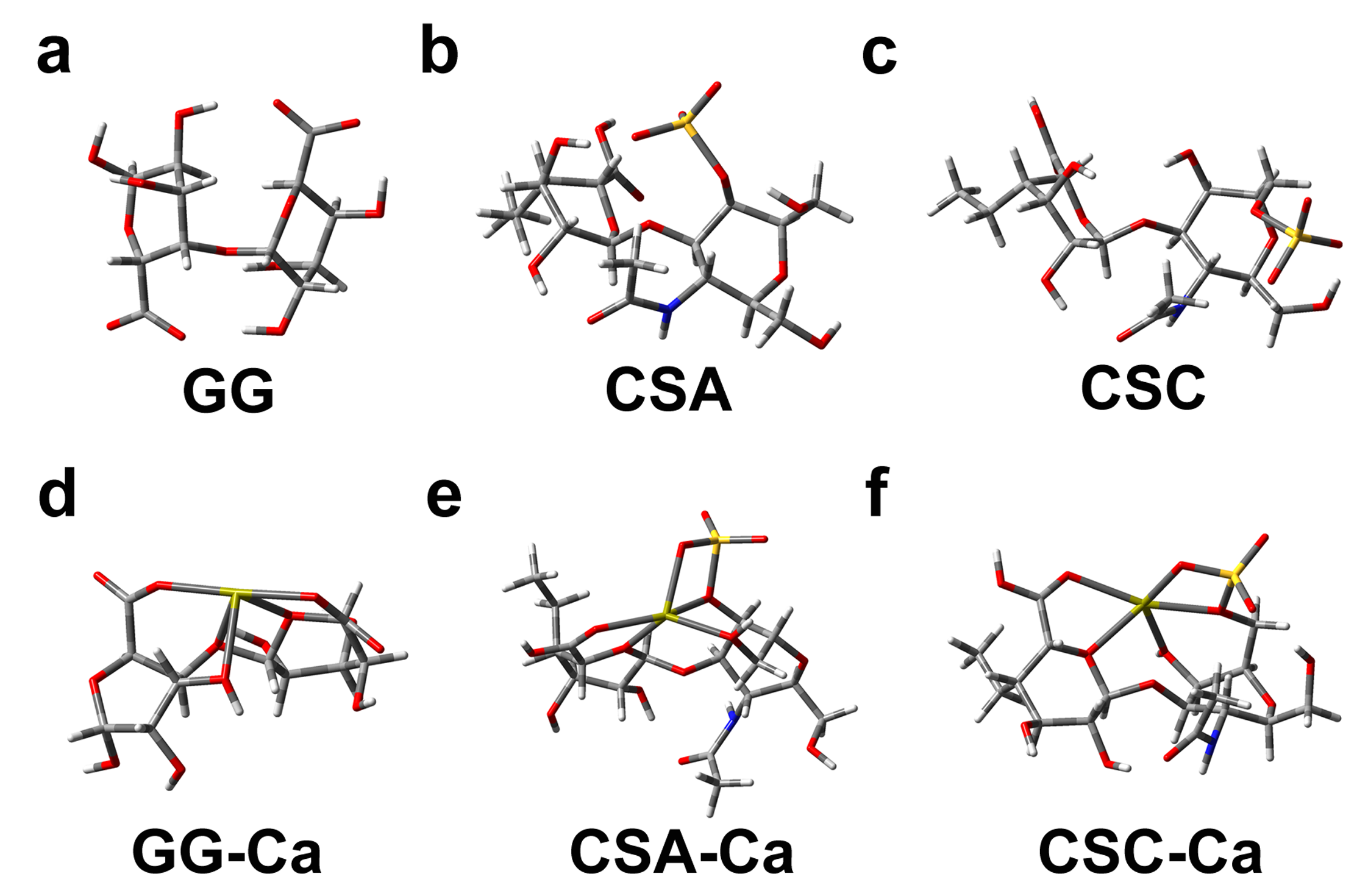


**Figure S6. Optimized structures for GG, CSA, CSC and the corresponding Ca^2+^ binding structures via the B3LYP method.** (**a**) Alginate molecule containing two L-guluronic acid residues (GG). (**b**) Chondroitin-4-sulfate molecule (CSA). (**c**) Chondroitin-6-sulfate molecule (CSC). (**d**) Binding structure of alginate-Ca^2+^ formed by one alginate chain composed of two G units and one Ca^2+^ (GG-Ca). (**e, f**) Binding structures of CS-Ca^2+^ formed by one CSA or CSC chain and one Ca^2+^ (CSA-Ca, CSC-Ca) (red color represents oxygen atom; blue color represents nitrogen atom; white represents hydrogen atom; grey color represents carbon atom; yellow represents sulfur atom; green represents calcium atom). Except for the alginate chain containing a pair of guluronate sequences, Ca^2+^ also can bind to CS (CSA and CSC) chain by the coordination reaction.

**Table S2.** Bond energies (in kcal/mol, 298 K and 1 atm) of calcium ions and GG, CSC and CSA calculated using the B3LYP, PBEPBE and M06-2X methods.

| **Methods** | **GG-Ca** | **CSC-Ca** | **CSA-Ca** | **GG-Ca-GG** | **GG-Ca-CSC** | **GG-Ca-CSA** |
| --- | --- | --- | --- | --- | --- | --- |
| **B3LYP/6-31G** | 463.59 | 350.20 | 338.91 | 555.40 | 546.88 | 534.23 |
| **PBEPBE/6-31G** | 459.58 | 347.51 | 337.96 | 553.48 | 544.14 | 529.93 |
| **M062X/6-31G** | 468.73 | 344.2 | 340.65 | 571.12 | 553.33 | 548.15 |


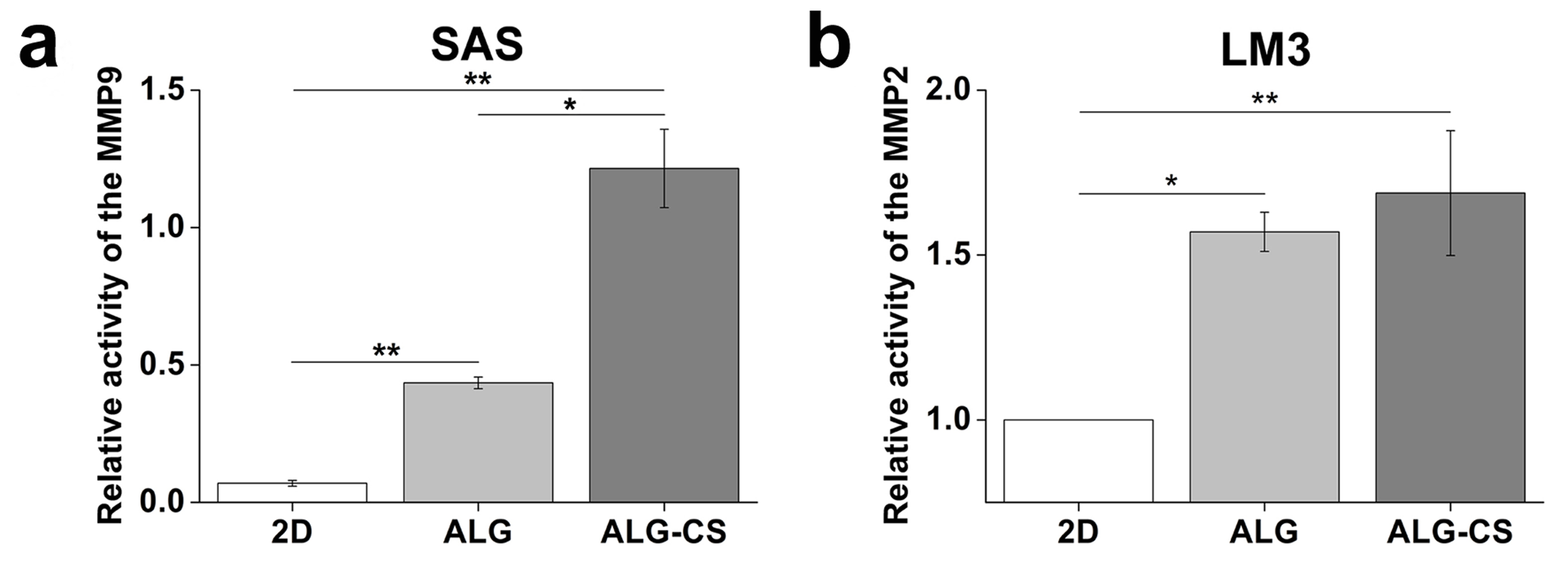


**Figure S7. Semi-quantitative analysis of MMP protein expression in SAS and LM3 cells cultured in beads (ALG and ALG-CS) according to the zymography results.** (**a**) SAS cells. (**b**) LM3 cells. 2D served as the control. Data are represented as the means ± SD of three independent experiments, *P<0.05, **P<0.01.


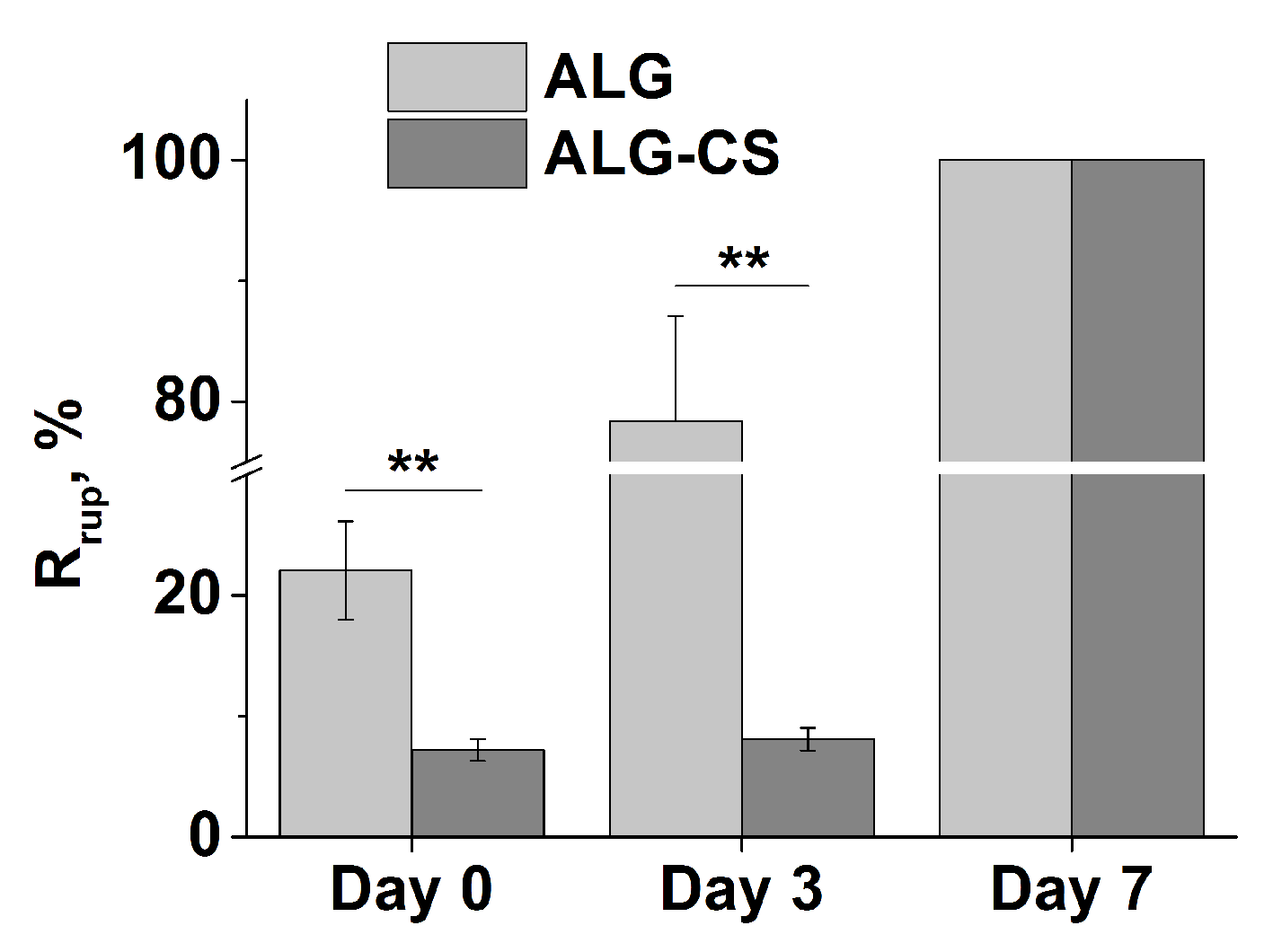


**Figure S8. Mechanical stability of ALG and ALG-CS soaked in cell culture medium at different time points.**


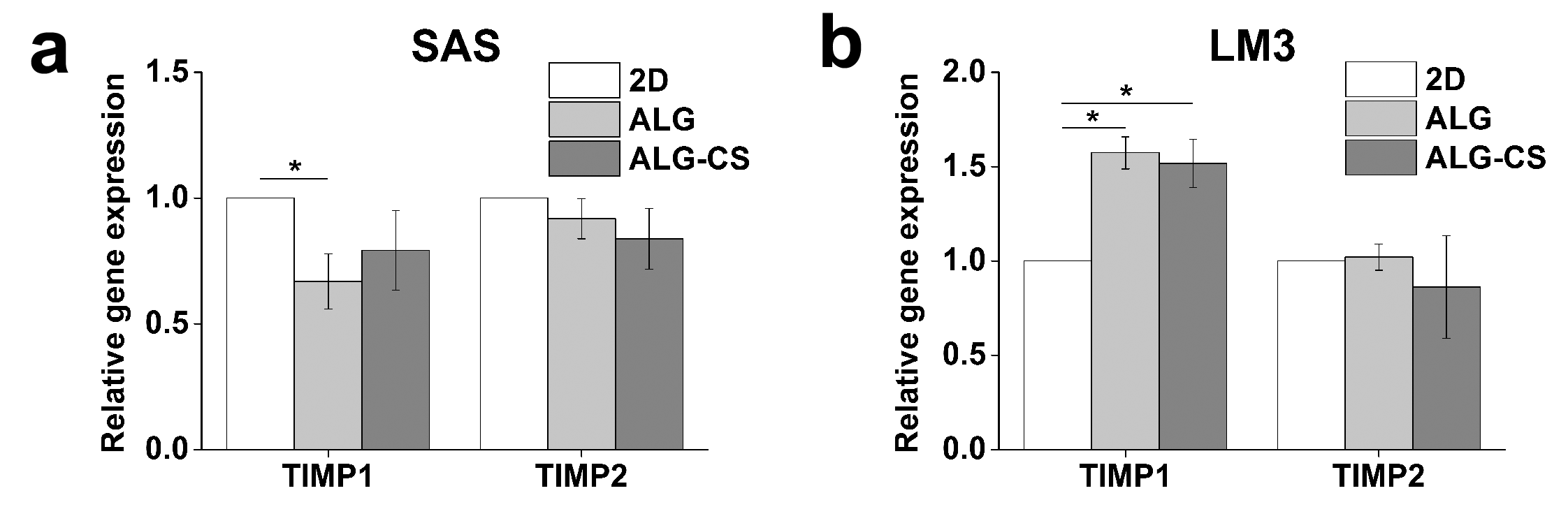


**Figure S9. Relative gene expression of TIMPs in SAS and LM3 cells cultured in both beads as detected by** **quantitative real-time PCR. *P<0.05.**

**Table S3.** Primer pairs used for quantitative real-time PCR studies.

| **Gene** |  | **Primer** |
| --- | --- | --- |
| **MMP2** | Forward  Reverse | 5’-CTCATCGCAGATGCCTGGAA-3’  5’-CAGCCTAGCCAGTCGGATTTG-3’ |
| **MMP9** | Forward  Reverse | 5’-TGGGCTACGTGACCTATGACAT-3’  5’-GCCCAGCCCACCTCCACTCCTC-3’ |
| **MMP14** | Forward  Reverse | 5’-GGAACCCTGTAGCTTTGTGTCTGTC-3’  5’-TGAGGGTCCTGCCTTCAAGTG-3’ |
| **TIMP1** | Forward  Reverse | 5’-CCTTATACCAGCGTTATGAGATCAA-3’  5’-AGTGATGTGCAAGAGTCCATCC-3’ |
| **TIMP2** | Forward  Reverse | 5’-GACGGCAAGATGCACATCAC-3’  5’-GAGATGTAGCACGGGATCATGG-3’ |
| **β-Actin** | Forward  Reverse | 5’-TGGCACCCAGCACAATGAA-3’  5’-CTAAGTCATAGTCCGCCTAGAAGCA-3’ |
